# Clinical effectiveness of decongestive treatments on excess arm volume and patient-centered outcomes in women with early breast cancer-related arm lymphedema: a systematic review

**DOI:** 10.11124/JBISRIR-2016-003185

**Published:** 2018-02-08

**Authors:** Eunice Jeffs, Emma Ream, Cath Taylor, Debra Bick

**Affiliations:** 1 Florence Nightingale Faculty of Nursing and Midwifery, King's College London, London, United Kingdom; 2 The Nottingham Centre for Evidence Based Healthcare: a Joanna Briggs Institute Centre of Excellence; 3 School of Health Sciences, University of Surrey, Guildford, United Kingdom; 4 Faculty of Life Sciences and Medicine, King's College London, London, United Kingdom

**Keywords:** breast cancer, decongestive lymphedema treatment (DLT), lymphedema

## Abstract

**Objective:**

To identify the effect of decongestive lymphedema treatment on excess arm volume or patient-centered outcomes in women presenting within either 12 months or a mean nine months of developing arm lymphedema following breast cancer treatment.

**Introduction:**

Lymphedema is a common consequence of breast cancer treatment requiring life-long treatment to reduce symptoms and prevent complications. Currently, evidence to inform the optimal decongestive lymphedema treatment package is lacking.

**Inclusion criteria:**

The review included studies on women who received lymphedema treatment within either 12 months or a mean of nine months of developing unilateral breast cancer-related arm lymphedema. The intervention was any decongestive lymphedema treatment delivered with the purpose of reducing arm lymphedema, compared to another form of lymphedema treatment (whether self or practitioner-administered), placebo or no treatment. The clinical outcome was excess arm volume; patient-centered outcomes were health-related quality of life, arm heaviness, arm function, patient-perceived benefit and satisfaction with treatment. Experimental study designs were eligible, including randomized and non-randomized controlled trials, quasi-experimental, prospective and retrospective before and after studies were considered.

**Methods:**

A three-step search strategy was utilized to find published and unpublished studies. The search identified studies published from the inception of each database to July 6, 2016. Reference lists were scanned to identify further eligible studies. Studies were critically appraised using appropriate standardized critical appraisal instruments from the Joanna Briggs Institute. Details describing each study and treatment results regarding outcomes of interest were extracted from papers included in the review using appropriate standardized data extraction tools from the Joanna Briggs Institute. Due to heterogeneity in included studies, results for similar outcome measures were not pooled in statistical meta-analysis. A narrative and tabular format was used to synthesize results from identified and included studies.

**Results:**

Seven studies reporting results for outcomes of interest were critically appraised and included in the review: five randomized controlled trials and two descriptive (uncontrolled) studies. Reported outcomes included excess arm volume (five studies), health-related quality of life (three studies), arm heaviness (one study), arm function (two studies) and patient-perceived benefit (two studies). There was some evidence that decongestive treatments were effective for women presenting within either 12 months or a mean of nine months of developing breast cancer-related arm lymphedema, but the wide range of data prevented comparison of treatment findings which limited our ability to answer the review questions.

**Conclusions:**

Weak evidence (grade B) for the impact of decongestive lymphedema treatment on women with early lymphedema (i.e. less than 12 months duration of BCRL symptoms) did not allow any conclusions to be drawn about the most effective treatment to be offered when these women first present for treatment. Findings provided no justification to support change to current practice.

Future primary research needs to focus on the most effective treatment for women when they first present with lymphedema symptoms, e.g. treatment provided within 12 months of developing symptoms. Studies should be adequately powered and recruit women exclusively with less than 12 months duration of breast cancer-related lymphedema symptoms, provide longer follow-up to monitor treatment effect over time, with comparable treatment protocols, outcome measures and reporting methods.

## Introduction

Lymphedema is a common sequelae of breast cancer treatment, causing arm swelling in around 20% of women following axillary node dissection.^1^ For example, of the 54,833 cases of female breast cancer diagnosed in the UK in 2014,^2^ it is likely that around 7000 of these women had developed arm swelling. Breast cancer-related lymphedema (BCRL) of the arm is a chronic tissue swelling arising from damage to lymph nodes and vessels, which may affect the ipsilateral arm and/or trunk, and women are considered to be at increased life-time risk of developing arm lymphedema following breast cancer treatment.^3-6^ Breast cancer-related lymphedema has significant physical, functional and psychological impact on the individual woman, with challenges for work, social and leisure activities, and potential financial implications.^5-10^

Lymphedema is a progressive condition, moving from an acute phase of subclinical edema (International Society of Lymphology/ISL grade 0), through mild or transient swelling, to chronic swelling with irreversible changes (ISL stage III).^11,12^ There is no agreement regarding the diagnostic threshold for lymphedema. At stage I, lymphedema may reduce with limb elevation, although by stage II tissue changes are occurring and pitting edema is present which no longer reduces with elevation alone; stage IIa indicates that excess fat and fibrotic changes are becoming established in the limb and pitting of edema may no longer be achieved; at stage III, pitting may be absent due to skin thickening, fatty deposits and increased fibrosis leading to characteristic changes of elephantiasis.^12^ The rate of progression is unknown but will vary between individuals, however chronic changes are likely to occur within 12 months of the onset of swelling.^13^ What is known is that without effective treatment, significant tissue changes can occur over time resulting in chronic, more severe lymphedema with tissue fibrosis which is less responsive to treatment and associated with increased morbidity.^11,14^ There is some evidence that the damage cannot be reversed once advanced lymphedema is established.^15^

The goal of lymphedema treatment is decongestion of the arm, that is, removal of excess lymph and associated tissue changes, to return the arm to a latent (hidden) phase of swelling. The improved swelling can then be managed by the individual with little personal or specialist input,^12,14^ although BCRL requires life-long self-management to reduce and control symptoms, prevent development of complications, and prevent recurrence of symptoms once the latent phase is achieved.^14,16^

Treatment of lymphedema aims to decongest the swollen limb and generally combines a form of compression (whether bandaging, hosiery or pneumatic compression therapy) with different forms of exercise (resistive, sequential; land or water-based), lymphatic massage, skin care and advice, and support for the patient;^12,14,16^ other treatments such as laser therapy have been used, and microsurgery and liposuction have increased in recent years.^12^ The internationally accepted current recommended best practice for lymphedema treatment is a two-phase decongestive lymphedema treatment (DLT), also commonly known as complex decongestive therapy (CDT).^12,14,16,17^ The first “intensive treatment” phase aims to decongest the swollen arm through two or more weeks of daily therapist-delivered treatment including multi-layer compression bandaging and manual lymph drainage (MLD). This is followed by a “maintenance” phase of patient self-treatment, with compression usually in the form of hosiery.^14^ There is some evidence of the effectiveness of DLT to reduce lymphedema,^17-20^ although studies to date have largely been underpowered, varied in their treatment protocol and assessment methods, and lack sufficient duration of follow-up (i.e. at least six months) to demonstrate any sustained treatment effect for this chronic condition.^17,21,22^

Assessment and monitoring of lymphedema generally include some objective measurement of size, which may be combined with self-assessment of health-related quality of life and symptoms, such as arm heaviness and arm function.^14,23^ Excess arm volume is the difference between the volume of swollen and non-swollen arms, and takes into account any changes to the whole body (such as body weight) by monitoring impact on the unaffected arm; change may be reported as an increase or decrease in excess arm volume.^14^ However, there is no agreement regarding the preferred methods for reporting changes following lymphedema treatment.

A search of the Cochrane Database of Systematic Reviews and Medline database using the keywords “lymph^∗^edema”, “breast cancer” and “review” was undertaken to establish the existence of any published systematic review and/or protocols for review regarding the population of interest. In the past six years, published systematic reviews have addressed the effectiveness of lymphedema treatment programs for women with BCRL^17-20,22,24-27^ and individual treatment modalities; for example, low level laser therapy (LLLT),^28-30^ intermittent pneumatic compression therapy (IPCT),^20,31,32^ and manual lymph drainage (MLD).^33,34^ However, no systematic review or protocol was found, and although Shah *et al.*^19^ reviewed evidence on the effectiveness of early detection and intervention to reduce the incidence of BCRL they did not focus on the effectiveness of treatment for lymphedema once symptoms are established. There is a need to specifically address the treatment of early lymphedema, where the skin and tissues are most likely to be responsive to treatment. For the purpose of this review, treatment is any intervention applied with the intent to decongest the swollen arm and early lymphedema is considered to be within 12 months of developing symptoms.^35,36^

This systematic review provides lymphedema practitioners, women with BCRL, and other decision makers with the first synthesis of available evidence for the effect of decongestive lymphedema treatment on excess arm volume and/or patient-centered outcomes when provided within either 12 months or a mean of nine months of symptoms developing. To guide the completion of this systematic review, a research protocol was designed and published in the Joanna Briggs Institute Database of Systematic Reviews and Implementation Reports.^37^

## Review question

The objective of the review was to identify the effect of decongestive lymphedema treatment on excess arm volume and patient-centered outcomes for women presenting within either 12 months or a mean of nine months of developing a swollen arm due to breast cancer-related lymphedema (BCRL). The specific review questions to be addressed were:

- What is the most effective combination of treatment elements for these women?
- What is the optimal duration of treatment?

## Inclusion criteria

### Participants

This review considered studies that included women with unilateral BCRL of the arm who received lymphedema treatment within 12 months of developing arm swelling. Although progression of lymphedema will vary between women, it is reasonable to assume that sufficient skin and tissue changes will have occurred by 12 months to affect the outcome of treatment. The original intention was to exclude from this review women with *more than* 12 months duration of symptoms. However, due to a lack of studies where the duration of BCRL symptoms was *less than* 12 months, a decision was made to also consider studies if the average (mean) duration of swelling was less than nine months. This time frame was considered likely to maximize the inclusion of studies with a large proportion of women with BCRL of *less than* 12 months duration but limit the number of women with chronic lymphedema (i.e. more than one year duration). Studies were also considered if outcomes were separately reported for a subgroup of women with *less than* 12 months duration; outcomes for women with more than 12 months duration were excluded.

Studies which reported participants with other forms of lymphedema (e.g. leg lymphedema, breast/truncal edema) were only included if data were separately reported for arm BCRL. Studies which included bilateral lymphedema or individuals defined as “at risk” of developing BCRL were excluded. Studies which included women with BCRL receiving concurrent cancer treatment (whether curative or palliative), with the exception of hormone therapy, were excluded as cancer treatment such as chemotherapy or radiotherapy could exacerbate BCRL through inflammation or increased fluid load.^38^ Men with BCRL were excluded from the review as the incidence of male breast cancer is less than 1%.^2^

### Interventions

Studies were considered that evaluated any conservative non-drug treatment where the goal was to decongest the arm, that is, reduce lymphedema, whether delivered by lymphedema therapist or patient self-management. The review was as inclusive as possible to capture all forms of decongestive treatment. The studies included, but were not limited to, the combination of treatments known as decongestive lymphedema treatment or DLT, complex/complete decongestive treatment or CDT; compression therapy, whether bandaging, garments or pneumatic compression pump; exercise, such as resistance training or hydrotherapy; low level laser therapy; manual lymph drainage or lymphatic massage.

Studies evaluating surgical or drug therapy interventions, treatment of progressive lymphedema due to uncontrolled active cancer, safety assessment of treatment, interventions used without the intention of lymphedema decongestion, evaluation of a single session of treatment (such as compression bandaging or hosiery, manual lymph drainage or exercise), interventions to reduce the risk of developing BCRL, or assessment techniques were excluded.

### Comparators

Relevant experimental studies could compare outcomes with another form of lymphedema treatment (whether patient or lymphedema therapist administered), placebo or no treatment.

### Outcomes

The review considered studies that included the following clinical or patient-centered outcome measures:

- The clinical outcome of interest was excess arm volume. Studies were sought which expressed the outcome as a relative change in excess arm volume (that is, compared to the non-swollen arm), whether measured by water displacement, perometry or circumference measurements to calculate arm volume. In the absence of relative change in excess arm volume, the intention was to consider the relative change in tissue fluid measured by bioimpedance or tissue dielectric constant.
- The patient-centered outcomes of interest were health-related quality of life, sensation of heaviness in the swollen arm, arm function, patient-perceived benefit or satisfaction with treatment. Studies which used an appropriate validated assessment tool or, in the absence of a validated tool, visual analog scales were included.

Studies which did not report on either the relative change to arm size/tissue fluid volume, psychosocial or patient self-report outcome measure, or patient value of treatment were excluded.

### Types of studies

The review included experimental study designs: randomized controlled trials, non-randomized controlled trials and quasi-experimental studies. It also considered descriptive (uncontrolled) studies including before and after studies, whether prospective or retrospective.

## Methods

### Search strategy

Studies were identified using a three-step search strategy. Firstly, a limited search of MEDLINE, Embase and CINAHL was undertaken to identify key words and index terms used in the title and abstract. Further scoping searches were used to identify the search terms and refine them for maximum sensitivity and specificity, to identify appropriate databases to ensure a thorough search of the relevant literature, and to ensure comparable search strategies across each chosen database. Database search terms were limited to variants of “breast cancer” and “lymphedema” in title and abstract fields, and reviews of the lymphedema literature, as attempts to refine the search by including additional treatment-related keywords led to the loss of potentially relevant papers. Secondly, a systematic search across all relevant electronic bibliographic databases using all identified variants of key words and index terms was undertaken to identify published and unpublished quantitative studies; the final search strategy (Appendix I) was deliberately left open with the expectation that eligibility criteria would be applied at the screening stage to a large number of references. Thirdly, reference lists and bibliographies of retrieved articles were reviewed and four specialist lymphology journals hand-searched to identify research studies not located through other search strategies.

Using the search strategy (Appendix I), the following databases were searched without date restrictions, on July 6, 2016 (except where specified), to identify studies published in the commercial literature (i.e. black literature): Allied and Contemporary Medicine (AMED), Biomed Central (search completed to July 1, 2015 as original search strategy could not be replicated in July 2016 due to changes to the database search function), BIOSIS (records were only available for the period 1969 to 2008 due to subscription limitations), British Nursing Index, CINAHL, Cochrane Library (Wiley Online Library), Embase, HMIC, MEDLINE, Physiotherapy Evidence Database (PEDro), PsycARTICLES, PsycINFO, PubMed, Scopus, Turning Research into Practice (TRIP) (search completed to July 1, 2015 as records obtained in July 2016 could no longer be downloaded to EndNote database without additional subscription), Web of Science and WorldCat. Preliminary searches of MEDLINE and PubMed identified many duplicate references but each produced some references which were not included by the other database, so both databases were included in the final search.

The following databases were searched on July 4, 2016 to identify studies conducted but not published in the commercial literature (i.e. gray literature): ClinicalTrials.gov (USA), Controlled Trials Register (ISRCTN), Grey Literature Report (http://www.greylit.org/ and www.opengrey.eu), International Clinical Trials Registry Platform (ICTRP), WorldCat ArticleFirst (OCLC), WorldCat Dissertations, WorldCat PapersFirst and WorldCat ProceedingsFirst.

The following databases were included in the review protocol search strategy but, following preliminary searches, were excluded from the final search strategy for the reasons specified: PROSPERO, Centre for Reviews and Dissemination reports only protocols for systematic reviews so not a source for primary research; DARE database is no longer maintained, but DARE records are included within the Cochrane Library; Clinical Trials Registers/UK Clinical Research Network (UKCRN) is now the Central Portfolio Management System which draws from two databases in the search strategy, i.e. ClinicalTrials.gov, ISRCTN; Conference Proceedings Citations Index (Web of Science) was included in the search of Web of Science Core Collection; Evidence NHS UK (NICE) searches databases included in the search strategy, i.e. AMED, British Nursing Index, CINAHL, Embase, HMIC, MEDLINE and PsycINFO; National Guideline Clearing House (USA) reports summaries of evidence and searches databases included in the search strategy, i.e. PubMed, Embase, CINAHL, the Cochrane Library, PsycINFO.

Online hand-searching of the titles and abstracts of all available editions of four lymphedema-specific journals took place on July 6, 2016: *European Journal of Lymphology*, *Journal of Lymphoedema*, *Journal of Phlebology and Lymphology* and *Lymphology*. In addition, the reference lists of published systematic reviews^17-22,24,27-34,39-53^ identified in the search were examined for additional references.

The review considered English and non-English language publications which provided an English language abstract. The development of current lymphedema treatment originated in continental Europe so no language restrictions were applied to the searches. The search of relevant foreign language full text papers to confirm eligibility for inclusion was conducted with the help of colleagues and online translation tools. As there were no eligible non-English language publications, it was not necessary to obtain a full translation of any paper.

The results obtained from each database search were electronically imported into EndNote X7 citation manager (Thomson Reuters, USA) and combined into a single library. Duplicate records were recorded and removed prior to screening.

All studies identified during the database search were assessed against the review eligibility criteria based on the information provided in the title and abstract. Studies identified from searching the reference lists were assessed for relevance based on the study title. A full report was retrieved and assessed for relevance for all studies which met the inclusion criteria or had insufficient detail in the abstract to determine eligibility.

### Assessment of methodological quality

The methodological quality of studies selected for inclusion was independently assessed by two reviewers (EJ and DB) using standardized critical appraisal instruments from the Joanna Briggs Institute System for the Unified Management, Assessment and Review of Information (JBI SUMARI), namely the Randomized Control/Pseudo-randomized Trial tool and Descriptive/Case Series Study tool.^54^

A decision was made not to exclude relevant papers on grounds of methodological quality, but to report the limitations. The reviewers (EJ and DB) resolved through discussion any differences of opinion that arose regarding methodological quality; the input of a third independent reviewer (ER) was not required.

### Data extraction

Details describing each study and results for the outcomes of interest were extracted from included papers using the standardized data extraction tool from JBI SUMARI.^54^ In addition to extraction of results for outcomes relevant to the review objectives and questions, extracted information included details about the interventions, populations and method of the included studies. Where studies reported outcomes for a subgroup of women with BCRL duration of *less than* 12 months or a mean of nine months, data was not extracted for those women with either *more than* 12 months or a mean of nine months BCRL duration.

### Data analysis and synthesis

Outcomes were expressed as continuous data, with means and standard deviation extracted from the included papers, where available, and analyzed. Due to wide study heterogeneity, the results for similar outcomes were not pooled in statistical meta-analysis. Instead, a narrative and tabular format have been used to display the results of this review.

Two subgroup comparisons were planned for: i) previous lymphedema treatment (yes or no), and ii) severity of swelling at baseline, whether mild or moderate-severe, where reported and as defined within the study. However, sub-group analysis could not be performed due to lack of data.

## Results

### Study inclusion

**Figure 1:**
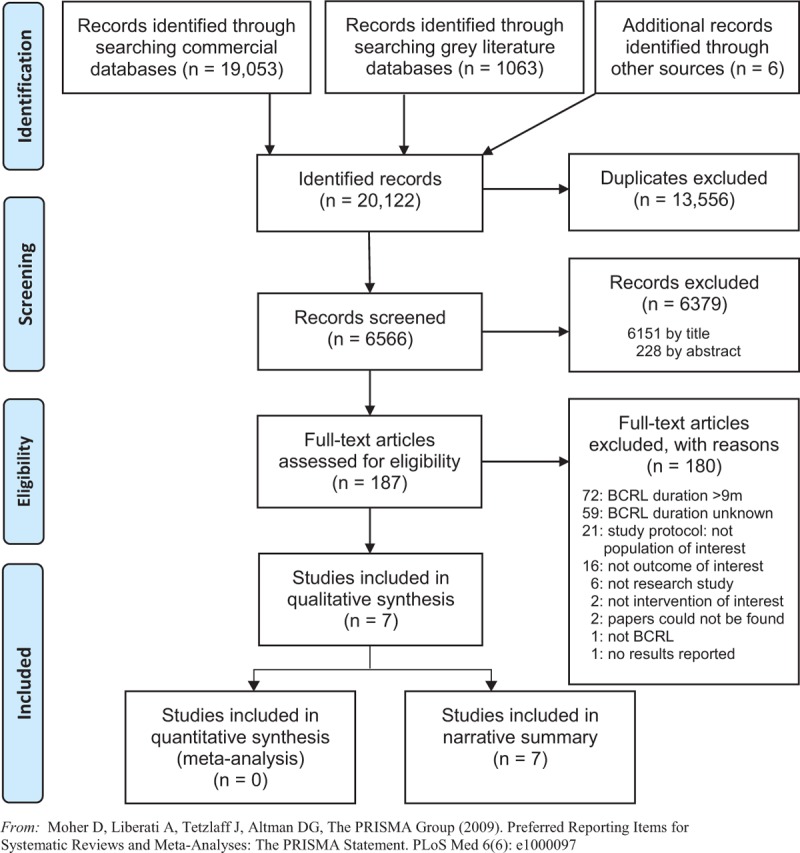
PRISMA flow diagram study selection process

Figure 1 presents an overview of the search and selection process in the form of a PRISMA diagram.

A total of 20,122 references were identified from the search for commercially published and gray literature. After removal of duplicate records and screening by title and abstract, 187 records remained for full text review, of which two papers^55,56^ could not be obtained via interlibrary loan. Authors of six conference abstracts were contacted to clarify eligibility criteria although none responded; 23 records of conference presentations could not be screened as they lacked an abstract, and four further records of conference presentations were excluded as they lacked corresponding author details to clarify missing data relating to eligibility criteria. Twenty-one study protocols were excluded, primarily because the study samples were not the population of interest for this review. Information about the duration of lymphedema symptoms was lacking in 66 published papers, of which 47 were published within the previous 10 years, so corresponding authors were contacted for to clarify whether they could report outcomes for either the study sample or a subgroup with *less than* 12 months or a mean of nine months BCRL duration. Responses were received regarding 24 papers, of which 21 papers were excluded as duration of BCRL was unknown^57-66^ or greater than 12 months/a mean of nine months,^67-74,76-78^ and three papers^35,36,75^ met the inclusion criteria. Appendix II lists the 180 studies excluded following full text examination and reasons for their exclusion.

Seven studies^35,36,75,79-82^ were critically appraised and included in the review regardless of their methodological limitations.

### Methodological quality

The results of the quality assessment using the JBI SUMARI appraisal tools for randomized controlled/pseudo-randomized trials and for descriptive (uncontrolled) studies are presented in Tables 1 and 2. Two^35,36^ of the five controlled studies and one descriptive study^79^ may be described as moderate to high quality, whereas the other studies^75,80-82^ were rated as having poor methodological quality.

#### Controlled studies

There were significant areas of bias in all study methods described.

Five studies were randomized controlled trials, however only two^35,36^ provided descriptions of the computer-generated randomization method demonstrating true random allocation with allocation concealed from allocator, whereas three^80-82^ merely stated that participants were randomized.

In only two studies^35,36^ were the assessors blinded to treatment allocation; the other three studies^80-82^ did not report whether the assessor was blinded (Q5, Table 3 ), meaning that risk of detection bias is unknown. Only one study^81^ used an intervention that could be blinded to both participants and assessors, although the method of providing sham laser was not reported. The other four studies^35,36,80,82^ could not disguise the study interventions so participants were not blind to treatment allocation.

Three studies^36,80,81^ reported comparable intervention and control groups at entry to the study, although sample sizes were small, ranging from four to 57 participants in each study, which affects the precision of estimates and increases the possibility of failure to detect an effect that is present (type II error).

One study,^80^ with no attrition, reported outcomes on an intention to treat basis. The other four studies^35,36,81,82^ reported reasons for attrition: two studies^36,81^ reported outcomes for only those who completed the study; one study^82^ reported data without citing the number of participants represented in the data; one study^35^ reported participant data available for each reported time-point.

Outcomes were measured in the same way for both intervention groups in all five controlled studies.^35,36,80-82^ As already noted, two of these studies^80,81^ did not measure in a reliable way all reported outcomes of interest so these data were not included in this review.

#### Descriptive studies

One uncontrolled retrospective case review study^79^ had low risk of bias. Possible bias in the other prospective study^75^ included insufficient detail to ensure reproducibility of reported outcome measurements; no follow-up of study outcomes following the immediate post-intervention period; and self-reported symptoms monitored using a tool that had not been validated.

### Characteristics of included studies

The key characteristics of the seven included studies^35,36,75,79-82^ are described below and, where appropriate, separately treated as controlled and descriptive/uncontrolled studies. Appendix III provides a summary of the characteristics by study.

#### Types of studies

Five studies were randomized controlled trials: two^35,36^ provided descriptions of the computer-generated randomization method, whereas three^80-82^ merely stated that participants were randomized. The final two papers were uncontrolled studies: one prospective before and after study^75^ and one retrospective case review.^79^

#### Participants and setting

No study specifically addressed the population of interest, although three studies^35,36,75^ reported one or more outcomes for a subgroup (n = 132) of women with BCRL duration ≤1 year from a total study sample of 282 women. Two studies^80,81^ (n = 48) reported outcomes for the total study sample of women with a mean duration of no more than 9 months (pooled mean 5.08 months; pooled SD 11.64), and two studies^79,82^ reported outcomes for a subgroup (one of the intervention groups) with a mean duration of no more than 9 months (mean 8.5 ± 6.6 and 8.3 ± 7.2 months respectively, n = 57) from a total study sample of 108 women. Five studies^35,79-82^ (n = 150) reported mean age with a pooled estimate of 54.70 ± 9.88 years; two studies^36,75^ reported age only for the total population, including women with >12 months duration BCRL.

Only two studies each recruited more than 50 women with *less than* 12 months duration of BCRL symptoms^35,75^ (n = 58 and 60). The number of participants with either *less than* 12 months BCRL duration or group mean ≤9 months duration of symptoms ranged from 8 to 40 in the other five studies.^36,79-82^

Breast cancer-related lymphedema was defined in the five controlled studies by arm size difference, but not in either descriptive study,^75,79^ using various diagnostic criteria recognized by the lymphedema community:^83^ two studies^35,81^ defined lymphedema as >10% excess arm volume and one study^82^ as >20% excess arm volume; one study^36^ defined BCRL as >150 ml difference between arms; one study^80^ described >2 cm circumference difference between arms. Four studies categorized severity of swelling at baseline, either according to the International Society of Lymphology staging,^12,82^ or relative (%) excess arm volume:^35,36,79^ Dayes *et al.*^35^ categorized severity as 10% to <20%, 20% to <30%, and ≥30% excess arm volume, McNeely *et al.*^36^ categorized severity as mild (<15% excess arm volume), moderate (16–37%), or severe (>37%), and Hwang *et al.*^79^ stratified severity as <20% or ≥20% excess arm volume. Two studies^75,82^ explicitly stated that participants had not received any treatment for BCRL prior to the intervention, whereas the other five studies^35,36,79-81^ reported neither the number of women who had previously received treatment nor the type of prior treatment received.

Six studies^35,36,75,79-81^ were performed in Lymphedema Units or Rehabilitation Departments within cancer services in developed countries with well-established health care systems; one study^82^ did not describe the setting. Two studies^35,36^ were conducted in Canada, two^75,81^ in Iran, two^79,80^ in Korea, and one^82^ in Poland.

#### Interventions and comparators

There was heterogeneity of treatment method and protocols. A summary of the interventions and protocols tested in the studies is presented in Table 3 , with more detail provided in appendices III and IV. Only two studies^36,82^ provided sufficient detail to replicate the method of intervention; the other five studies^35,75,79-81^ require the reader to make assumptions regarding aspects of the treatment provided. Four studies^35,36,75,82^ specified the lymphology school where the therapists had trained. Two studies^79,80^ did not specify the method of MLD used.

***Complex decongestive treatment***

Complex decongestive treatment utilizes therapist-delivered multi-layer compression bandaging and MLD in the first/intensive phase of treatment, followed by a maintenance phase of self-treatment with compression hosiery in an effort to maintain the long-term benefits of CDT.^12^

Five studies addressed the benefit of CDT: three controlled studies^35,80,82^ each utilized a different CDT protocol and comparator, and two uncontrolled studies^75,79^ also varied the protocol used.

Dayes *et al.*^35^ compared three weeks of phase 1 CDT with self-treatment, phase 2 CDT, with both groups carrying out self-treatment for the remainder of the study protocol; this study provided the only comparison of intensive therapist treatment versus patient self-treatment to decongest the arm. Gradalski *et al.*^82^ compared two weeks of phase 1 CDT with compression bandaging alone, followed by six months of daily self-treatment (phase 2 CDT) for both groups with flat-knit hosiery and continuation of the exercise program and skin care. Kim *et al.*^80^ also utilized CDT although, as the focus of the study was to determine the effect of adding active resistive exercise to CDT, this study is reported below under the heading of exercise.

Haghighat *et al.*^75^ prospectively reviewed the effect of 10–15 sessions of phase 1 CDT over a two-three week period; the protocol did not include a period of phase 2 CDT. Hwang *et al.*^79^ conducted a retrospective review of patients who received two weeks phase 1 CDT followed by 24 months of self-treatment (phase 2 CDT) with compression hosiery, self-MLD, remedial exercises and skin care, plus night-time self-bandaging three times per week.

***Compression bandaging and manual lymph drainage (MLD)***

One controlled study^36^ sought to ascertain whether four weeks treatment with multi-layer compression bandaging alone was sufficient to effect a reduction in lymphedema or whether the additional cost of therapist-provided MLD could be justified by enhanced treatment outcomes. The study protocol did not include a post intervention self-treatment or maintenance phase.

***Exercise***

Exercise is considered a standard part of CDT, both during the intensive therapist phase and the subsequent self-treatment maintenance phase. Five studies^35,75,79,80,82^ reported exercise as part of the study protocol, however, only one controlled study^80^ specifically examined the therapeutic role of exercise to decongest the swollen arm. Kim *et al.*^80^ compared CDT plus active resistive exercise with CDT alone, with both intervention groups receiving two weeks identical CDT comprising application of daily compression bandages and MLD, and self-treatment with daily remedial exercises and breathing exercises; this was followed by a maintenance phase of six weeks self-CDT. The CDT plus active resistive exercise group undertook an additional daily 15 minutes resistive exercise throughout the two weeks CDT and six weeks self-CDT.

One controlled study^82^ reported that an identical unspecified exercise regime was carried out by both groups, and another controlled study^35^ reported that exercise was carried out by both groups but did not specify the regime or whether both groups undertook the same exercise regime. The two uncontrolled studies^75,79^ included unspecified exercises as part of the CDT regime.

***Low level laser therapy***

The treatment of lymphedema with low level laser therapy is thought to have a multi-factorial effect to increase lymph flow and thereby reduce tissue fluid and proteins.^84^ One study^81^ compared the effect of light therapy (890 nm wavelength) to placebo, that is, sham laser. Both groups received three times weekly treatment for three weeks (18 sessions); the intervention group received one Joule energy to each of five points in the axilla of the affected arm, whereas the sham laser group did not receive any laser energy. A further three weeks (18 sessions) of active treatment or sham laser took place after an eight week rest period. No concurrent treatment was reported for either group.

#### Outcomes

A summary of the outcomes assessed in the studies is presented in Table 4. Only one study^36^ reported training the assessors. Of the three corresponding authors^35,36,75^ who were contacted for clarification of information specific to outcomes for women with <12 months duration of BCRL, one author^35^ supplied all the requested data, one^75^ supplied some of the requested data, and the other^36^ reported the data were no longer available to them.

The most common outcome measure used to evaluate CDT was excess arm volume: five^35,36,75,79,82^ of the seven studies reported changes in excess arm volume. Three standard measurement methods were used to calculate volume: water displacement;^36,75^ Perometry,^79^ an opto-electronic measurement machine; and manual circumference measurements,^35,36,82^ using the formula for a truncated cone to calculate arm volume. Studies^23,85,86^ have shown these three measurement methods to be equally valid, producing comparable but not interchangeable limb volume data. Volume changes were variously reported in terms of pre and post intervention excess arm volume^82^ pre and post intervention percentage excess arm volume,^35,79^ post intervention percentage reduction in excess arm volume,^35,36,75^ and post intervention relative change in excess arm volume^82^ using the formula proposed by Ancukiewicz *et al.*;^87^ McNeely *et al.*^36^ reported outcomes for the population of interest only in graph format. Relative change, whether reported as percentage reduction in excess arm volume compared to baseline^23^ or using the formula for ratio of ratios proposed by Ancukiewicz *et al.*,^87^ takes into account variations within the individual and between different women such as the impact of body mass on arm size. The arm volume data reported by the two other studies^80,81^ were excluded from the review as one^81^ calculated arm volume using sum of circumferences, a method not included in the review, and the other^80^ reported only the volume of the affected arm; absolute volume does not take into account the contralateral (unaffected) arm volume or changes that occur in both arms. None of the studies^35,36,75,79-82^ measured bioimpedance spectroscopy or tissue dielectric constant.

Health-related quality of life was reported in two^35,80^ of the seven studies using the 36-item Short Form Health Survey (SF-36; RAND, USA). The SF-36^88,89^ is a set of eight self-reported quality of life measures and has been widely used to assess the impact of a health intervention on different components of an individual's health; summary scores are used to derive physical and mental health component scores, ranging from 0 to 100, with a higher score indicating better overall function. It should be noted that, although commonly used in BCRL research, SF-36 does not address specific symptoms suffered by BCRL patients such as heavy and swollen arms. Only one study^82^ used a lymphedema-specific quality of life tool, although this Lymphedema Questionnaire was not a validated tool.

One study^81^ reported on heaviness in the affected arm using a 10-cm visual line, however, data were not reported as average scores but instead represented in graph format without clear labelling of the axes. Another study^75^ assessed heaviness with a self-rating scale of 0–3, with 0 indicating no symptoms and 3 indicating severe symptoms, but did not provide a subgroup analysis for women with *less than* 12 months duration of BCRL symptoms. Data for this outcome were excluded from the review.

Two studies^35,81^ reported arm function using two different methods. One study^81^ used a visual analog scale with a 10-cm line to measure participant perception of range of motion, although data were not reported as average scores but instead represented in graph format without clear labelling of the axes. The other study^35^ used the Disabilities of the Arm Shoulder and Hand questionnaire (DASH). These functional tools are not comparable.

Only two studies^81,82^ addressed patient-perceived satisfaction with treatment: Kaviani *et al.*^81^ used a visual analog scale with a 10 cm line to determine the participant's desire to continue with low level laser therapy; Gradalski *et al.*^82^ used a numerical rating scale to determine satisfaction with treatment by assessing quality of life (0 = least to 10 = best quality of life).

Wide heterogeneity in study populations, interventions and measurement outcomes made meaningful comparison difficult and meant meta-analysis could not be conducted.

#### Length of follow-up

Despite the chronicity of lymphedema and potential for treatment failure post intervention,^90^ the long-term impact of the intervention was reported by only three of the seven studies: Gradalski *et al.*^82^ monitored participants for six months post intervention, Dayes *et al.*^35^ monitored for one year and Hwang *et al.*^79^ for two years. The other four studies^36,75,80,81^ did not monitor the effect of treatment beyond the completion of the intervention period.

## Review findings

The findings from the seven included studies^35,36,75,79-82^ are presented below, reported according to intervention for the primary outcomes of interest relating to excess arm volume and patient-centered outcomes. Results for each outcome are presented only for subgroups of participants with reported BCRL duration of *less than* 12 months^35,36,82^ and intervention groups where mean BCRL symptom duration is no more than 9 months;^79-81^ no study recruited only women with BCRL symptoms duration *less than* 12 months.

### Complex decongestive therapy

Four studies^35,75,79,82^ (n = 152) reported some immediate post intervention reduction in excess arm volume; the other study^80^ involving CDT did not measure relative excess limb volume. Table 5 presents the reduction as absolute excess arm volume (ml)^82^ or relative (%) excess arm volume,^35,79^ and Table 6 presents the relative (%) change in excess arm volume;^35,75,82^ long-term follow-up data are also shown for one study.^35^

Two controlled studies^35,82^ (n = 79) reported significant post intervention reduction in excess arm volume: Dayes *et al.*^35^ reported a greater post intervention reduction in excess arm volume for the self-treatment group (42% ± 57, n = 23) compared to the CDT group (28 ± 47, n = 31), although there was no significant between-group difference in reduced excess arm volume (*p*>.05) at 12 months follow-up; Gradalski *et al.*^82^ reported a significant post intervention reduction in excess arm volume for the CDT group (46%, n = 25) which was retained at six months follow-up. One uncontrolled study^75^ (n = 60) reported (by personal communication) a 46% post intervention reduction in excess arm volume, whereas the other^79^ (n = 32) reported an unchanged relative arm volume at six months follow-up for women with a baseline of less than 20% excess arm volume. The limited available data precluded subgroup comparison by severity of BCRL symptoms.

Only two studies^35,79^ provided long-term follow-up, with one controlled study^35^ reporting retention of intervention benefit and further improvement in both groups over a 12-month period, and the other uncontrolled study^79^ reporting a non-significant increase in percentage excess arm volume over the subsequent two-year period; a third study^82^ reported retention of treatment benefit at six months post intervention, however there was no statistically significant difference to the other intervention group (compression bandaging) whose outcomes are not reported here as duration of BCRL symptoms was a mean of 9.4 ± 10.2 months.

Gradalski *et al.*^82^ (n = 25) reported a statistically significant post intervention improvement in lymphedema-specific quality of life of 1.69 points (on a 10-point scale), although the clinical meaningfulness of this is unknown. One study^35^ (n = 54) reported no post intervention improvement in health-related quality of life in either group, although below average health status (SF-36 summary scores<50) was recorded pre and post intervention for both the whole study sample (n = 98) and the subgroup of women with <12 months duration of BCRL (n = 54). Another study^80^ (n = 40) reported a statistically significant post intervention improvement in SF-36 scores in both groups with a greater improvement in the CDT plus active resistive exercise group compared to CDT alone (*p*<.05). The findings are presented in Tables 7 and 8.

Dayes *et al.*^35^ reported non-significant improvement to arm function (DASH scores) in both groups. The other study^81^ assessing function did not report average self-rating scores as average scores.

Data for the effect of treatment on heaviness in the affected arm were not included in this review: one study^81^ did not report data as average scores and the other^75^ reported data only for the whole study sample including women with *more than* 12 months BCRL duration.

A high level of satisfaction with treatment was reported by one controlled study^82^ (n = 25) although the between group difference was not statistically significant: the CDT group scored 9.4, with 10 representing highest satisfaction with treatment, and the compression bandaging group scored 8.8.

### Compression bandaging and manual lymph drainage

McNeely *et al.*^36^ (n = 18) reported in graphical format a post intervention percentage reduction in excess arm volume for women with ≤11 months BCRL duration: 56% reduction for the combined intervention and 47% for compression bandaging alone; this reduction was significantly greater than that achieved by women with ≥1 year BCRL duration (*p* < 0.05).

### Exercise

Kim *et al.*^80^ (n = 40) reported only one relevant outcome of interest: health-related quality of life using SF-36 summary scores to derive physical and mental health component scores (Tables 7 and 8); they also measured arm volume but reported only the treated (ipsilateral) arm volume, so the findings were not considered by this review.

Kim *et al.*^80^ recorded a statistically significant improvement in both groups for the components of physical and mental health; the intervention group showed a greater improvement in physical and mental health (9 and 5 extra points respectively) compared to the control group, but the clinical significance of this improvement is not reported. However, it should be noted that Ware *et al.*^88^ reported SF-36 summary measures with confidence intervals of ± 6–7 points, which suggests that the SF-36 findings reported by Kim *et al.*^80^ may not be clinically significant. Participants in this study^80^ reported better pre-intervention health status (score 65) than Dayes *et al.*^35^

### Low level laser therapy

Kaviani *et al.*^81^ (n = 8) reported women's self-rating of arm heaviness and desire to continue with low level laser therapy in graphical format which lacked explanatory detail. The graphs appear to record a slight increase in arm heaviness for laser intervention and slight reduction in arm heaviness with sham laser, and a willingness to continue the laser intervention but not sham laser.

In summary, there is some evidence (grade B) that decongestive treatments effectively reduce lymphedema in women with early BCRL (i.e. duration of <12 months or a mean of nine months), whether provided as CDT or compression bandaging with or without MLD (see Table 9). As each study reported a different treatment protocol, this review was not able to determine the most effective combination of treatment elements to reduce excess arm volume or improve patient-centered outcomes for women with BCRL duration of less than 12 months. There was weak evidence (grade B) for the addition of active resistive exercise to enhance the benefit of CDT. Evidence (grade B) for the benefit of low level laser therapy or intermittent compression therapy for this population is inconclusive. Evidence was lacking for the long-term benefit of decongestive treatment for this population, and the heterogeneity of the treatment protocols precludes the comparison of treatment findings and thus identification of the most effective treatment for this population.

## Discussion

The aim of this review was to identify the effect of decongestive lymphedema treatment on excess arm volume and patient-centered outcomes for women presenting within either 12 months or a mean of nine months of developing a swollen arm due to BCRL. A key challenge was the limited number of published studies that have measured and reported results of treatment for women with early BCRL. Although it is likely that earlier treatment will result in better outcomes,^12,91-93^ this review cannot recommend what form this treatment should take. Furthermore, no study focused specifically on this group of women, nor did any published protocol for ongoing or recently completed studies refer to the recruitment of women within 12 months of developing BCRL symptoms.

Two-phase CDT is considered by clinical experts^12,14,94^ to be the recommended treatment for lymphedema, supported by the findings of a review of systematic reviews.^27^ This review found some evidence for the effect of CDT to reduce arm volume, as four studies^35,36,75,82^ reported a statistically significant post intervention reduction; however, Dayes *et al.*^35^ reported a significantly greater arm volume reduction with self-treatment on completion of the three-week intervention period, although there was no statistically significant difference in volume reduction between the groups at six weeks or 12 months. To date, Dayes *et al.*^35^ is the only randomized controlled trial to compare an intensive therapist-delivered treatment phase with self-treatment to decongest a swollen arm, with findings contrary to clinical expectations of a greater improvement with CDT. Further research is necessary to determine whether women could benefit from costlier intensive treatment or achieve similar outcomes with more convenient and less resource intense self-management. The recent move towards surveillance of women following breast cancer treatment, with the potential to introduce treatment at a pre-clinical stage,^19^ does not preclude a need to determine the most effective – and cost-effective – treatment for women who present with clinical symptoms of BCRL.

As with previous reviews,^17,21,22,27^ we found heterogeneity of interventions and assessment methods, and variations in quality of study design and reporting, which limited comparison of results. The variation in treatment protocols emerged as a major limitation of the review, with no comparative protocols identified to address the two aspects of the review question: the most effective treatment elements and optimal duration of treatment. Despite the chronicity of lymphedema and potential for treatment failure post intervention reported elsewhere,^90^ most researchers did not report the long-term impact of the intervention or treatment failure. The review findings support those of previous reviews^17,21,22,27^ that studies are underpowered and lack sufficient follow-up.

The priority outcomes and measurement methods which are meaningful to women and practitioners have yet to be determined,^27^ although a survey of 421 Australians with different types of lymphedema found that 60% of participants considered reductions in swelling, heaviness and tightness, and improvement in range of movement to be very important treatment outcomes.^95^ The effect of treatment has primarily been measured by changes to limb volume, however there was no consensus regarding a standardized format to ensure that a comparison could be made between different protocols; this is reflective of the published research into lymphoedema treatments.^94^ Similarly, there was wide variation in the patient-centered outcomes measured and methods used. Although the statistical significance of findings was reported for some study results, the importance of these findings for patients and practitioners was not reported; the minimal clinically important change is not known, either for objective measures such as changes in arm volume or subjective measures of changes to patient symptoms or quality of life.

A strength of this review was the inclusivity of literature from a wide range of databases with no restriction to date or language of publication. It is possible that small but eligible studies may have been published in journals that do not provide an English abstract, although additional resources would be required to search databases where there is no English abstract. No studies were found which recruited only women with no more than 12 months duration of BCRL symptoms, which was a limitation of the review. However, data were obtained from three studies with published outcomes for subgroups of women with BCRL duration of *less than* 12 months^35,36,75^ and, where it was not possible to access raw data to determine actual duration of BCRL, an inclusion criteria of mean BCRL duration of ≤9 months likely limited the number of participants with >1 year duration of BCRL.^79-82^

## Conclusion

There was weak evidence (grade B) for the impact of decongestive lymphedema treatment for women with early BCRL (i.e. duration of <12 months or a mean of nine months). It was not possible to identify the optimal treatment components to reduce excess arm volume or improve patient-centered outcomes for these women, nor to determine the optimal duration of treatment. The lack of comparable treatment protocols across studies did not allow any conclusions to be drawn about the most effective treatment to be offered to women when they first present for lymphedema treatment.

### Recommendations for practice

There was no evidence to justify change to current practice.

### Recommendations for research

Future primary research needs to focus on the most effective, acceptable and cost-effective treatment for women when they first present with BCRL symptoms, that is, treatment provided within 12 months of developing symptoms. Studies should be adequately powered and recruit women exclusively with less than 12 months duration of BCRL symptoms so that true inferences about the population of interest can be drawn from the results obtained.

A consistent method of reporting outcomes is necessary to permit comparison between studies and meta-analysis in order to build the evidence base. Change in excess arm volume should be considered a key outcome measure and reported as a percentage of baseline excess arm volume. Collaborations should be encouraged between groups of lymphedema researchers, particularly with regard to creating comparable treatment protocols, outcome measures and reporting methods. Inclusion of women with BCRL in designing studies could help to identify priority outcomes and select appropriate patient reported outcome measures.

Studies should include longer follow-up times to monitor the benefit of treatment in the months following completion of the intervention. Lymphedema is a chronic condition and studies should identify the proportion of participants achieving treatment failure as well as those achieving sustained success.

## Acknowledgements

The reviewers acknowledge the support of the University of Nottingham Centre for Evidence Based Healthcare: a Joanna Briggs Centre of Excellence.

The authors would like to thank Trevor Murrells, statistician at the Florence Nightingale Faculty of Nursing, Midwifery and Palliative Care, King's College London, for his statistical advice and calculations, and also the following for their feedback on the final draft of this report: Arnie Purushotham, Professor of Breast Cancer, King's College London; Margaret Sneddon, Senior Research Fellow, University of Glasgow; Rachel Rawson, Senior Clinical Nurse Specialist, Breast Cancer Care; and Sarah Walsh, breast cancer patient.

## Funding

Eunice Jeffs is funded by a National Institute for Health Research (NIHR/HEE) Clinical Doctoral Research Fellowship (CDRF-2013-04-023).

This report is part of independent research funded by the National Institute for Health Research. The views expressed in this publication are those of the authors and not necessarily those of the NHS, the National Institute for Health Research or the Department of Health.

## Figures and Tables

**Table 1:** Results of critical appraisal for appraising randomized controlled / pseudo-randomized trials

Citation	Q1Random allocation	Q2Patients blinded	Q3 Allocator blinded	Q4ITT analysis	Q5Assessor blinded	Q6Groups comparable	Q7Treated equally	Q8Same outcomes	Q9Reliably measured	Q10 Appropriate statistical analysis	Total
Dayes *et al.* 2013^35^	Y	N	Y	N	Y	N	Y	Y	Y	Y	7/10
Gradalski *et al.* 2015^82^	Y	N	?	N	?	?	Y	Y	?	?	3/10
Kaviani *et al.* 2006^81^	?	Y	?	N	?	Y	?	Y	N	?	3/10
Kim *et al.* 2010^80^	N	N	?	?	?	Y	Y	Y	N	Y	4/10
McNeely *et al.* 2004^36^	Y	N	Y	N	Y	Y	Y	Y	Y	Y	8/10
%	60	20	40	0	40	60	80	100	40	60	

? = unclear; N = no; N/A = not applicable; Y = yes.

**Table 2:** Results of critical appraisal for appraising descriptive studies

Citation	Q1Random allocation	Q2Clearly defined inclusion criteria	Q3 Confounding factors accounted for	Q4Objective assessment	Q5Description of compared groups	Q6Appropriate follow-up time	Q7Withdrawals accounted for	Q8Reliable outcome measures	Q9Appropriate statistical analysis	Total
Haghighat *et al.* 2013^75^	N	Y	Y	?	N/A	N	?	Y	Y	3/7
Hwang *et al.* 2013^79^	N	Y	Y	Y	Y	Y	N/A	Y	Y	6/8
%	0	100	100	50	100	50	0	100	100	

? = unclear; N = no; N/A = not applicable; Y = yes.

**Table 3:** Interventions in included studies

Study/citation	Components of intervention	Duration and frequency of intervention	Comparator	Outcomes measured	Measurement method
**Complex decongestive treatment (CDT/CDPT/DLT)**					
Dayes *et al.* 2013^35^^,^^a^	Intensive phase: MLD and short stretch compression bandaging (Vodder or Foldi method); advice re skin care, exercise, and maintenance of healthy body weight.Followed by maintenance phase: elastic compression garments; exercise, skin care, maintenance of healthy body weight.	5× per week for 4 weeks (20 sessions).Subsequent maintenance treatment with daily self-care	Compression therapy: daily self treatment, as per maintenance phase, see below	1. EAV2. HRQOL3. Limb function	1. Manual circumference measurement2. Short Form Health Survey (SF-36)3. Disabilities of the Arm Shoulder and Hand (DASH) questionnaire
Gradalski *et al.* 2015^82^^,^^b^	Multi-layer compression bandaging and Vodder II method MLD, exercise and deep diaphragmatic breathing.Followed by maintenance phase: exercise, compression garment, arm and skin care.	5× per week for 2 weeks (10 sessions). Followed by 6 months daily self-care.	Compression bandaging 5× per week for 2 weeks (10 sessions), followed by 6 months maintenance phase of daily self-care, see below.	1. EAV2. HRQOL3. Patient-perceived treatment benefit	1. Manual circumference measurement2. non-validated lymphedema questionnaire3. VAS: desire to continue treatment
Haghighat *et al.* 2013^75^^,^^a^^,^^c^	CDT phase I: Vodder method MLD; multi-layer short-stretch bandages; remedial exercise (not specified); skin care.	5× per week for 2–3 weeks (10–15 sessions).	None	1. EAV2. Symptom of arm heaviness	1. Water displacement2. VAS
Hwang *et al.* 2013^79^^,^^b^^,^^d^	CDT phase I: 1 hour MLD, multi-layer low stretch compression bandaging, remedial exercises, skin care.CDT phase II: compression garments daytime, self-bandage night-time; self-MLD/self-massage; exercise; skin care.	5× per week for 2 weeks (10 sessions).Then daily self-treatment plus 3 times per week night-time self-bandaging.	None	1. EAV	1. Perometry
Kim *et al.* 2010^80^^,^^b^	Termed ‘Complex Decongestive Physiotherapy (CDPT)’Intensive phase: MLD, compression therapy, exercise, breathing exercises.Maintenance phase: self-CDPT	5× per week for 2 weeks (10 sessions). Then 6 weeks self-administered CDPT.	Complex Decongestive Therapy plus Active Resistive Exercise (CDPT+ARE), see below	1. HRQOL	1. SF-36
**Complex decongestive physiotherapy (CDPT) and active resistive exercise (ARE)**					
Kim *et al.* 2010^80^^,^^b^	Intensive phase: MLD, compression bandage, exercises and breathing exercises, plus Active Resistive Exercise (ARE) program using 0.5 kg dumbbell.Maintenance phase: self-CDPT+ARE using 1 kg dumbbell (if tolerated), wearing hosiery.	5× per week for 2 weeks (10 sessions).Then 6 weeks self-administered CDPT+ARE.	CDPT, see above	1. HRQOL	1. SF-36
**Compression bandaging (CB) and manual lymph drainage (MLD)**					
McNeely *et al.* 2004^36^^,^^a^	Short stretch bandages plus 45 minutes daily Vodder method MLD. Education re arm care and skin care.	5× per week for 4 weeks (20 sessions)	CB 5× per week for 4 weeks (20 sessions), see below	1. EAV	1. Water displacement & manual circumference measurement
**Compression bandaging (CB)**					
McNeely *et al.* 2004^36^^,^^a^	Short stretch bandages. Education re arm care and skin care.	5× per week for 4 weeks (20 sessions)	CB+MLD 5× per week for 4 weeks (20 sessions), see above	1. EAV	1. Water displacement & manual circumference measurement
**Compression therapy**					
Dayes *et al.* 2013^35^^,^^a^	Elastic compression garments, advice re skin care, exercise and maintenance of healthy body weight.	Daily self-care with compression garments worn 12 hours per day	CDT, 5× per week for 4 weeks (20 sessions). Subsequent maintenance treatment with daily self-care, see above	1. EAV2. HRQOL3. Limb function	1. Manual circumference measurement2. SF-363. DASH questionnaire
**Low level laser therapy (LLLT)**					
Kaviani *et al.* 2006^81^^,^^b^	Laser diode (product specified), output power 10 Watts at 890 nm wavelength. Treatment to five points in axilla at 1 cm distance from skin, energy density 1.5J/cm^2^	3x per week for 3 weeks, then 8 week break, then 3x per week for 3 more weeks. (18 sessions).	Sham irradiation as per LLLT, total 18 sessions. Double-blinded conditions for sham laser not specified.	1. Symptom of arm heaviness2. Limb function3. Patient-perceived treatment benefit	1. VAS2. Goniometry: range of movement3. VAS: desire to continue treatment

EAV, excess arm volume; HRQOL, health-related quality of life; MLD, manual lymph drainage; VAS, visual analogue scale.

^a^Subgroup with BCRL duration <12 months.

^b^Mean duration ≤9 months.

^c^Prospective before and after (uncontrolled) study.

^d^Retrospective case review (uncontrolled) study.

**Table 4:** Outcomes measured in included studies

Study/citation	Domain	Outcomes measured	Approach
Haghighat *et al.* 2013^75^McNeely *et al.* 2004^36^	Change in excess arm volume	Excess arm volume	Water displacement
Hwang *et al.* 2013^79^			Perometer
Dayes *et al.* 2013^35^Gradalski *et al.* 2015^82^McNeely *et al.* 2004^36^	Manual circumference measurement
Dayes *et al.* 2013^35^Kim *et al.* 2010^80^	Health-related quality of life	Health-related quality of life	Short Form Health Survey (SF-36)
Gradalski *et al.* 2015^82^	Health-related quality of life	Lymphedema Questionnaire (non-validated)
Haghighat *et al.* 2013^75^Kaviani *et al.* 2006^81^	Symptom of heaviness	Sensation of heaviness in affected arm	Visual Analogue Scale (VAS), subjective reporting by patient
Kaviani *et al.* 2006^81^	Limb function	Range of movement	VAS, subjective reporting by patient
Dayes *et al.* 2013^35^	Disabilities of the arm, shoulder and hand	Disabilities of the Arm, Shoulder and Hand (DASH) Questionnaire
Gradalski *et al.* 2015^82^Kaviani *et al.* 2006^81^	Patient-perceived benefit or satisfaction with treatment	Desire to continue treatment	VAS, subjective reporting by patient

**Table 5:** Results for studies reporting excess arm volume (ml)

Study	Method	Number of participants (n)	Pre-intervention EAV	Post-interventionEAV	Final reportedEAV
***Complex decongestive treatment (CDT/DLT)***					
Dayes *et al.* 2013^35^^,^^a^**DLT group**^b^^,^^c^**Control group**^b^^,^^c^	Manual circumference measurement	3123	23% ± 1221% ± 7	17% ± 12^e^12% ± 12^e^	15% ± 15^e^15% ± 13^e^
Gradalski *et al.* 2015^82^**CDT group**^b^^,^^d^**CB group**	Manual circumference measurement	25^‡^	898 ± 445^‡^	472 ± 285^e^^,^^f^^‡^	506 ± 263^e^^‡^
Hwang *et al.* 2013^79^**CDT group 1 (<20%EAV)**^b^^,^^d^**CDT group 2 (≥20%EAV)**	Perometer	32^‡^	11% ± 5^‡^	10% ± 9^g^^‡^	14% ± 11^g^^‡^

EAV = excess arm volume.

^‡^Data not presented: mean breast cancer-related lymphedema (BCRL) duration >9 months.

^a^Data provided by corresponding author.

^b^Data presented as relative (%) EAV, i.e. 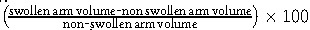.

^c^Subgroup of women with BCRL duration <12months.

^d^Mean BCRL duration ≤9 months.

^e^*p* > .05 between group difference.

^f^*p* < .05 within group difference.

^g^No *p* values reported.

**Table 6:** Results for studies reporting percentage reduction in excess arm volume

Study	Duration of intervention (weeks)	Number of participants (n)	Measurement method	Mean % EAV reduction post-intervention	Mean %EAV reduction at week 4–7	Mean %EAV reduction at 3 months	Mean %EAV reduction at 6 months	Mean %EAV reduction at 1 year
***Complex decongestive treatment (CDT/DLT)***								
Dayes *et al.* 2013^35^^,^^b^**DLT group**^c^**Control group**^c^	4	3123	Manual circumference	28 ± 47^e^42 ± 57^e^	29 ± 45^e^28 ± 25^e^	36 ± 35^e^38 ± 46^e^	45 ± 35^e^38 ± 34^e^	36 ± 64^e^37 ± 51^e^
Gradalski *et al.* 2015^82^**CDT group**^d^**CB group**	2	25‡	Manual circumference	Reported: 47% relative volume change^87^^,^^f^‡				
Haghighat *et al.* 2013^75^^,^^b^**CDT group**^c^	2–3	60	Water displacement	46 ± 13^g^	-	-	-	-
***Compression bandaging (CB) and manual lymph drainage (MLD)***								
McNeely *et al.* 2004^36^ ^‡^**CB+MLD group**^c^**CB group**^c^	4	810	Water displacement	56^‡^ ^g^^,^^h^47^‡^ ^g^^,^^h^				
810	Manual circumference	62^‡^ ^g^^,^^h^44^‡^ ^g^^,^^h^				

EAV = excess arm volume.

^‡a^Percentage reduction in EAV calculated as 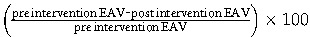.

^b^Data provided by corresponding author.

^c^Subgroup of women with BCRL duration <12months.

^d^Mean BCRL duration ≤9 months.

^e^*p* > .05 within group difference.

^f^*p* < .05 within group difference.

^g^No *p* values reported.

^h^Data calculated from published graph.

^‡^Data not presented: mean BCRL duration >9 months.

**Table 7:** Results for studies reporting Short Form Health Survey (SF-36) Physical Component summary score

Study	Number of participants (n)	Mean pre-intervention score	Mean post-intervention score
***Complex decongestive treatment (DLT)***			
Dayes *et al.* 2013^35^^,^^a^^,^^b^**DLT groupControl group**	3124	42.9 ± 7.544.6 ± 6.8	45.1 ± 7.3 (n = 39)^d^44.8 ± 6.3 (n = 22)^d^
***Complex decongestive physiotherapy (CDPT) and active resistive exercise (ARE)***			
Kim *et al.* 2010^80^^,^^c^**CDPT+ARE groupCDPT group**	2020	68.25 ± 17.4268.50 ± 11.01	85.12 ± 13.89^e^76.00 ± 12.73^e^

^a^Data provided by corresponding author.

^b^Subgroup of women with BCRL duration <12 months.

^c^Mean BCRL duration ≤9 months.

^d^*p* > .05 for all change score comparisons.

^e^*p* < .05 according to repeated-measures analysis of variance between pre and post treatment in each group.

**Table 8:** Results for studies reporting Short Form Health Survey (SF-36) Mental Component summary score

Study	Number of participants (n)	Mean pre-intervention score	Mean post-intervention score
***Complex decongestive treatment (DLT)***			
Dayes *et al.* 2013^35^^,a,^^b^**DLT groupControl group**	3124	43.7 ± 5.842.9 ± 6.0	43.9 ± 4.3 (n = 39)^d^44.9 ± 6.4 (n = 22)^d^
***Complex decongestive physiotherapy (CDPT) and active resistive exercise (ARE)***			
Kim *et al.* 2010^80^^,^^c^**CDPT+ARE groupCDPT group**	2020	66.25 ± 15.1264.25 ± 16.85	75.25 ± 14.73^e^69.50 ± 17.63^e^

^a^Data provided by corresponding author.

^b^Subgroup of women with BCRL duration <12 months.

^c^Mean BCRL duration ≤9 months.

^d^*p* > .05 for all change score comparisons.

^e^*p* < .05 according to repeated-measures analysis of variance between pre and post treatment in each group.

**Table 9:** Summary of individual study findings for women with breast cancer-related lymphedema symptom duration <12 months

Citation	Excess arm volume	HRQOL	Heaviness	Arm function	Patient benefit	Group difference *p*
***Complex decongestive treatment (CDT/DLT)***						
Dayes *et al.* 2013^35^^,^^a^**DLT group**^b^**Control group**^b^	YY	OO		OO		>.05
Gradalski *et al.* 2015^82^**CDT group**^c^**CB group**	Y^‡^	Y^‡^			Y^‡^	>.05^‡^
Haghighat *et al.* 2013^75^^,^^a^**CDT group**^b^	Y		Not reported			Not applicable
Hwang *et al.* 2013^79^**CDT group 1 (<20% ELV)**^c^**CDT group 2 (≥20% ELV)**	O^‡^					Not applicable
***Compression bandaging (CB) and manual lymph drainage (MLD)***						
McNeely *et al.* 2004^36^,**CB+MLD group**^b^**CB group**^b^	YY					Not reported
***Complex decongestive physiotherapy (CDPT) and active resistive exercise (ARE)***						
Kim *et al.* 2010^80^**CDPT+ARE group**^c^**CDPT group**^c^		YY				>.05
***Low level laser therapy***						
Kaviani *et al.* 2006^81^**Laser group**^c^**Sham group**^c^			??		??	Not reported

Y = improved; O = unchanged; N = worse; ? = data unclear.

^a^Data provided by corresponding author.

^b^Subgroup of women with BCRL duration <12months.

^c^Mean breast cancer-related lymphedema duration ≤9 months.

^‡^ = data not presented: mean BCRL duration >9 months.

## Appendix I: Search strategies for individual databases

The following databases were searched on July 6, 2016 (unless stated otherwise) to identify studies published in the commercial literature.

### WorldCat

The following databases were searched on 4^th^ July 2016 to identify studies conducted but not published in the commercial literature (i.e. gray literature):
